# A Mechanically Flexible Superhydrophobic Rock Wool Modified with Reduced Graphene Oxide‐Chloroperene Rubber for Oil‐Spill Clean‐Up

**DOI:** 10.1002/gch2.202100072

**Published:** 2021-09-05

**Authors:** Maryam Davardoostmanesh, Hossein Ahmadzadeh

**Affiliations:** ^1^ Department of Chemistry Faculty of Science Ferdowsi University of Mashhad Mashhad 9177948974 Iran

**Keywords:** chloroperene rubber, oil/water separation, reduced graphene oxide, superhydrophobic rock wool

## Abstract

The leakage of industrial oil and organic wastewater discharge has caused serious damage to the natural environment and ecology. Therefore, implementation of a low‐cost and high‐performance adsorbent material is of great significant. This work reports the preparation of superhydrophobic rock wool (RW) for efficient clean‐up of oil and organic solvents. The modified RW is prepared by coating a commercial RW with reduced graphene oxide (RGO) nanosheets under hydrothermal treatment. To improve the adhesion between the RGO nanosheets and RW, a film of chloroperene rubber is deposited on the RW surface followed by modification with RGO. The modified RW possesses superhydrophobicity and superoleophilicity with a water contact angle of 152.4°, and it is used for separation of oil–water mixture. The modified RW exhibits excellent mechanical elasticity and durability when compared with commercial one, and the adsorbed oils are recycled by simple squeezing. Its oil adsorption capacities are maintained above 95%, after several compression cycles. Importantly, the modified RW exhibits excellent photothermal properties which are beneficial for the separation of high‐viscosity oils. Owing to low costs, versatility, and scalability in production, the modified RW can be regarded as a suitable choice for large‐scale oil/water separation.

## Graphical abstract

Modified rock wool (RW) is prepared using low cost materials such as reduced graphene oxide and polychloroperene (CR). Addition of CR into the porous structure of RW improves roughness, flexibility, and durability. The modified RW has excellent photothermal properties which is beneficial for the separation of high‐viscosity oils under natural sunlight.

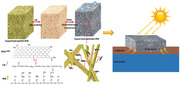

## 1 Introduction

Oil‐spill accidents and discharge of industrial wastewater have caused serious environmental issues. The spilled oil and contaminated water can be transported by ocean currents to large distances, creating permanent effects on the marine ecosystem. In the Sanchi oil spill accident in 2018, one of the most polluting oil spill accident into the 21st century, ≈136 000 tons of oil spilled into the East China.^[^ ^1^ ^]^ This accident imposed devastating impacts on the environment. In this regard, the cleanup strategies of oil spill should be implemented to significantly reduce the environmental damages. Conventional strategies including skimming,^[^ ^2^ ^]^ bioremediation,^[^ ^3^ ^]^ in situ burning,^[^ ^4^ ^]^ and adsorption^[^ ^5^ ^]^ have been widely used for oil–water separation. Among these approaches, adsorption is considered the most effective method with simplicity and low cost for oil pollutions removal.^[^ ^6^ ^]^ Several adsorbent materials such as carbon aerogels,^[^ ^7^ ^]^ graphene sponges,^[^ ^8^ ^]^ and magnetic foams^[^ ^9^ ^]^ have been used for this purpose due to their large pore volume and strong adsorption capacity. However, complicated preparation processes, high costs, and scalability challenges restrict the practical applications of these adsorbent materials. In this regard, the use of 3D porous materials such as wools and sponges is more effective.^[^ ^10^, ^11^ ^]^

Rock wool (RW) is an inorganic fiber material with the characteristics of high tensile strength, high chemical resistance, and excellent incombustibility.^[^ ^12^ ^]^ It has been widely used as thermal insulator in buildings and industrial equipment. RW can be a good candidate for oil/water separation owning to its porous structure, low density, and low cost. However, due to the mixed hydrophilic/oleophilic properties, the original RW adsorbs both water and oil simultaneously. Thus, it is not suitable for selective adsorption of oil. The modification of original RW could improve its hydrophobicity. Hao et al.^[^ ^13^ ^]^ recently used polydimethylsiloxane (PDMS)/silica nanoparticle as modifying agent for preparation of superhydrophobic RW. However, PDMS/silica nanoparticle used as modifier is quite expensive which limits its large‐scale applications.

Graphene materials including graphene and reduced graphene oxide (RGO) are promising candidates as modifier considering their hydrophobicity.^[^ ^14^, ^15^ ^]^ Carbon lattice of graphene and its derivatives efficiently repels polar molecules and enhances affinity towards oil and organic solvents.^[^ ^16^, ^17^ ^]^ Graphene materials can increase the roughness of a substrate when used as coating. Presence of small amounts of oxygen‐containing functional groups on RGO increases affinity toward polar solvents.^[^ ^16^ ^]^ Jamsaz and Goharshadi^[^ ^18^ ^]^ used polyurethane sponge coated with RGO and orthoaminophenol for oil–water separation. The prepared sponge exhibited the adsorption capacities of 80 times of its own weight for several oil and organic solvents. RGO can be produced in large scale and low price. The π‐electron delocalization on RGO leads to the broadband absorption of the solar spectrum.^[^ ^19^ ^]^ Thus, it can be heated up under natural sunlight to decrease oil viscosity and collect high‐viscosity spilled oil to facilitate the separation process. Wang et al.^[^ ^20^ ^]^ reported RGO nanosheets coated melamine sponge with excellent photothermal property. Upon light irradiation, the surface temperature of RGO‐melamine sponge increased rapidly and lowered oil viscosity.

In this work, for the first time, we used RGO‐coated RW for selective adsorption of oil from oil–water mixtures. However, modified RW with RGO can be degraded in high‐viscosity oils because of weak adhesion between RGO and RW surface and the poor mechanical stability of the rough surface structure. To further enhance hydrophobicity and recyclability, chloroprene rubber (CR) was deposited on the RW surface followed by the second modification with RGO. The elasticity of CR is crucial for preparation of recyclable modified RW. CR shows better mechanical properties than other resins and adhesive materials and allows fast oil recovery by simple squeezing method. Zulfiqar et al.^[^ ^21^ ^]^ created a robust superhydrophobic surface using CR as an adhesive to bind sawdust particles on glass slide and used it for oil/water separation. The introduction of CR layer to RW structure further enhanced the adhesion of RGO into the RW surface and increased the roughness, flexibility, and durability. The resulted RGO/CR‐RW composite possesses desirable hydrophobicity and oleophilicity.

## 2 Results and Discussion

### 2.1 Preparation

**Scheme 1:**
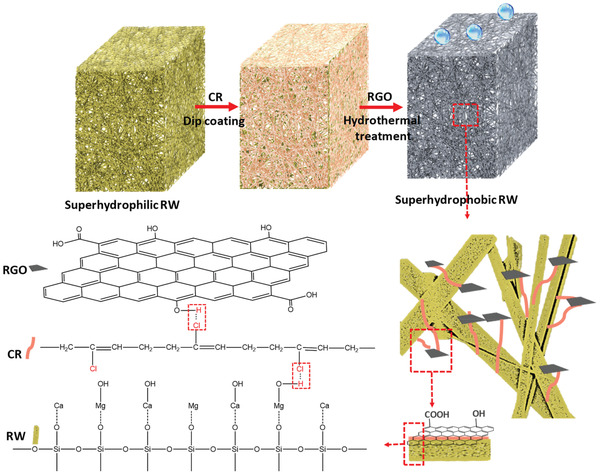
Schematic diagrams of preparation superhydrophobic RW.

The schematic diagrams of preparation superhydrophobic RW and the possible interactions are shown in **Scheme** **1**. To improve the mechanical properties and hydrophobicity, CR adhesive layer was deposited on the surface of the RW by a simple dip‐coating method. CR generates a layer of coating on the RW surface. Then, a secondary layer of RGO nanosheets was covered on the RW framework. The CR can bond RGO nanosheets onto the RW surface using H‐bonds via the interaction between the Cl atom and O of functional groups such as COOH and OH and enhances the bonding energy between RGO and RW.^[^ ^22^ ^]^ The hydrophobic RGO/CR composite created the hierarchical rough structure with low‐surface‐energy property on the RW surface. The hydrophobic carbon lattice of RGO could interact with a wide range of organic solvents, hydrocarbons and oils through π–π conjugation, while it efficiently repels polar molecules such as water. Also, it can increase the roughness of a surface when used as a coating and further enhance the surface hydrophobicity. The small amounts of oxygen containing functional groups present on RGO enhance the affinity toward polar solvents.

#### 2.1.1 Factors Affecting the Preparation of RGO/CR‐RW

To obtain an optimal preparation, we studied the effect of hydrothermal reduction time, hydrothermal reduction temperature, graphene oxide (GO) concentration, and CR concentration. The samples were prepared under different hydrothermal temperature (100, 200, and 400 °C) and different hydrothermal time (1, 2, and 4 h). The hydrophobicity of the modified RW is mainly related to the reduction degree of GO. In 100 °C, the modified RW had somewhat hydrophilic properties. In the low temperature, the oxygen‐containing functional groups on the GO surface were less removed. In addition, the high hydrothermal temperature (400 °C) affects the physical state of RW, thereby affecting the surface energy of RW. In addition, GO was not completely reduced in a short time. With increasing hydrothermal time, the degree of reduction of GO gradually increased and the prepared RW obtained proper hydrophobicity. Thus, appropriate hydrothermal condition for preparation of modified RW was 200 °C for 2 h.

Additionally, high concentration of GO on the RW structure might block the pores, leading to reduction of the adsorption capacity. The superhydrophobicity RW increased in presence CR due to the reduced surface energy, while excessive CR concentration did not contribute to the superhydrophobicity of RW. High concentration of CR led to a collapse of overall macrostructure of RW.

### 2.2 Characterization

**Figure 1:**
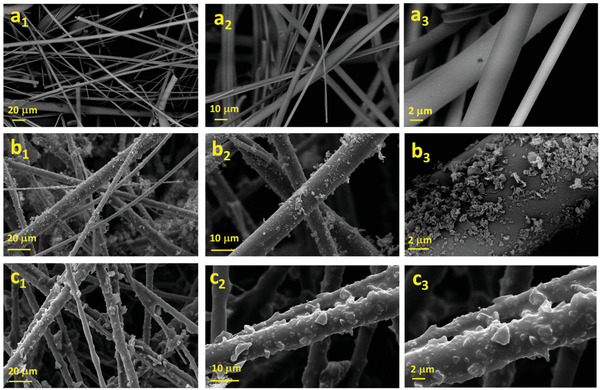
SEM images of a_1_– a_3_) RW; b_1_–b_3_) RGO‐RW; c_1_–c_3_) RGO/CR‐RW. Right and left columns show 20, 10, and 2 µm magnification, respectively.

The morphology of the pristine and the modified RW is presented in **Figure** **1**. The scanning electron microscopy (SEM) images of the pristine RW show a smooth structure (Figure 1a_1_–a_3_). Figure 1b_1_–b_3_ shows the modified RW with RGO nanosheets without CR layer, in which RGO nanosheets are heterogeneously adhered to the RW surface resulting in a relatively rough surface. High‐magnification SEM image (Figure 1b_3_) displays a large number of RGO sheets on the framework of the RW. However, there is not strong binding force between RGO nanosheets and RW surface, resulting in poor stability. Figure 1c_1_–c_3_ shows a thin layer of CR covered on the RW surface with strong adhesion the RGO nanosheets onto the RW surface. RGO nanosheets are embedded in CR layer provides hierarchical rough structure. The introduction of CR onto the RW structure further enhanced the roughness. This rough structure is due to the agglomeration of RGO nanosheets onto the RW surface under the adhesion of CR.

**Figure 2:**
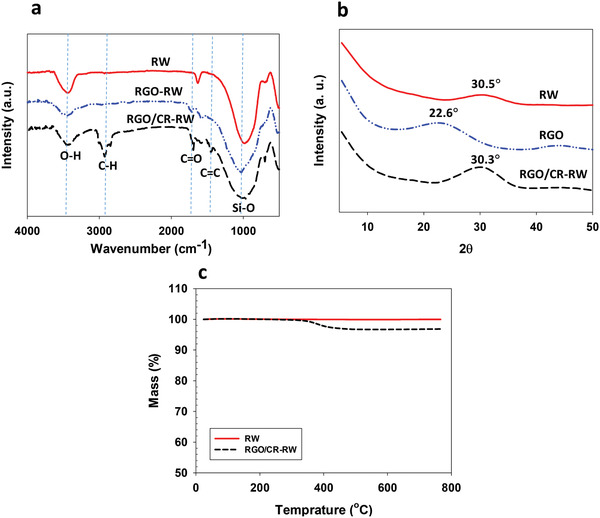
a) FTIR spectra of RW, RGO‐RW, and RGO/CR‐RW; b) XRD patterns of RW, RGO, and RGO/CR‐RW; c) TGA curves of RW and RGO/CR‐RW.

The Fourier transform infrared (FTIR) spectra of the modified and unmodified RW are depicted in **Figure** **2**a. Different steps of RW modifications are shown in this figure. The strong absorption band around 990 cm^−1^ is attributed to the existence of oxide compounds of Al, Mg, Ca, and Si on the surface of the RW.^[^ ^12^ ^]^ There is a weak band at 3460 cm^−1^ attributed to —OH groups on RW structure. These functional groups are both involved in adsorption of oil and water. After modification with RGO, the intensity of band corresponding to —OH decreased. The absorption bands at 1615 and 1704 cm^−1^ are assigned to the presence of stretching and bending vibrations of —C=C and —C=O groups, respectively. In the case of RGO/CR‐RW, the intense bands at 2900 and 1450 cm^−1^ are attributed to the stretching and bending vibrations of C—H groups in CR.^[^ ^23^ ^]^

To further confirm the successful modification of RW, the structural properties of both pristine and modified RW were also determined using X‐ray diffraction (XRD) analysis. As shown in Figure 2b, a characteristic peak (2θ = 30.5°) is found in the XRD pattern of RW, suggesting the existence of CaO, MgO, SiO_2_, and Al_2_O_3_ compounds.^[^ ^12^ ^]^ The XRD pattern of RGO shows a strong and sharp diffraction peak at 2θ = 22.6° and a relatively weak diffraction peak at 2θ = 43.8°.^[^ ^24^ ^]^ The XRD pattern of RGO/CR‐RW shows an intense characteristic peak at 2θ = 30.3°. It can be observed that the RGO/CR‐RW have diffraction pattern characteristics similar to RW, suggesting that modification have few effects on the crystalline structures of RW.

The thermal stability of the RW and RGO/CR‐RW were determined by TGA and is shown in Figure 2c. Obviously, the pristine RW had no clear weight loss in the temperature range of 25–800 °C indicating high thermal stability of the unmodified RW. The RGO/CR‐RW display only a weight loss of ≈3% at 300–500 °C, which is probably due to the decomposition of O‐containing functional groups in RGO and degradation of CR.^[^ ^25^, ^26^ ^]^ It can be concluded that presence of RGO/CR as modifier on the RW surface does not make a significant difference in thermal stability of RW.

### 2.3 Superhydrophobicity

**Figure 3:**
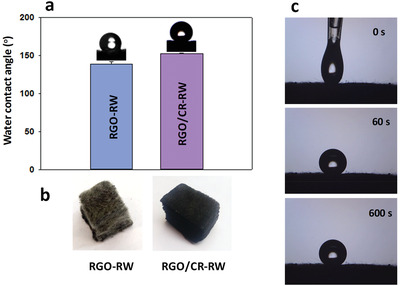
a) WCA of RGO‐RW and RGO/CR‐RW; b) images of RGO‐RW and RGO/CR‐RW; c) photographs of a water droplet on RGO/CR‐RW during 600 s exposure period.

The surface hydrophobic properties were investigated by measuring the WCA of the pristine and modified RW. The pristine RW showed superhydrophilicity with a WCA of 0°. The results also verified that the contact angle for directly grafting RGO onto the RW surface was only 138.5 ± 3.2°, which did not have superhydrophobic property. When the CR was coated on RW surface, the WCA was increased to 152.4 ± 0.2°, changing the property from hydrophobic to superhydrophobic surface (**Figure** **3**a). This can be attributed to the intensification of roughness obtained from RGO nanosheets when coupled with low surface energy of adhesive materials (Figure 1). This result confirms that the CR adhesive layer plays an important role in the preparation of superhydrophobic RW. As shown in Figure 3b, unlike the RGO‐RW, the RGO/CR‐RW composite turned black because of RGO nanosheets being completely coated on the RW surface. It should be noted that the water droplet could maintain spherical shape on the surface of modified RW for more than 600 s, exhibiting the high hydrophobicity of RGO/CR‐RW composite (Figure 3c).

**Figure 4:**
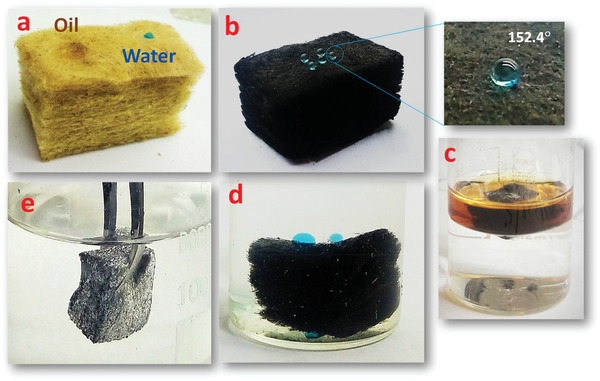
a) Hydrophilicity and oleophilicity of pristine RW; b) superhydrophobicity of RGO/CR‐RW; c) oleophilicity of RGO/CR‐RW; d) superhydrophobicity of RGO/CR‐RW in *n*‐hexane; e) mirror reflection of RGO/CR‐RW under water.

**Figure** **4**a shows that water and oil droplets are both immediately adsorbed onto the pristine RW (water dyed with methylene blue). The water droplets are spherical on the RGO/CR‐RW and easily roll off the surfaces, indicating the superhydrophobic nature of the modified RW (Figure 4b). Additionally, the modified RW exhibited not only superhydrophobicity but also superoleophilicity (Figure 4c). Figure 4d shows the water droplets readily departed from the RGO/CR‐RW surface immersed in *n*‐hexane (Video S1, Supporting Information). This indicated that the superhydrophobicity of RGO/CR‐RW composite was retained even in the presence of an organic solvent.

The superhydrophobic nature of modified RW was further verified by the formation of a silver mirror‐like surface when RGO/CR‐RW immersed in water using an external force (Figure 4e). This appearance is attributed to entrapping air between the water and surfaces of the adsorbent which prevents water from entering the pores.^[^ ^27^ ^]^ These results showed that the RGO/CR‐RW is not only desirable oil‐adsorbent but also possess oil–water separation ability.

### 2.4 Mechanical Property

**Figure 5:**
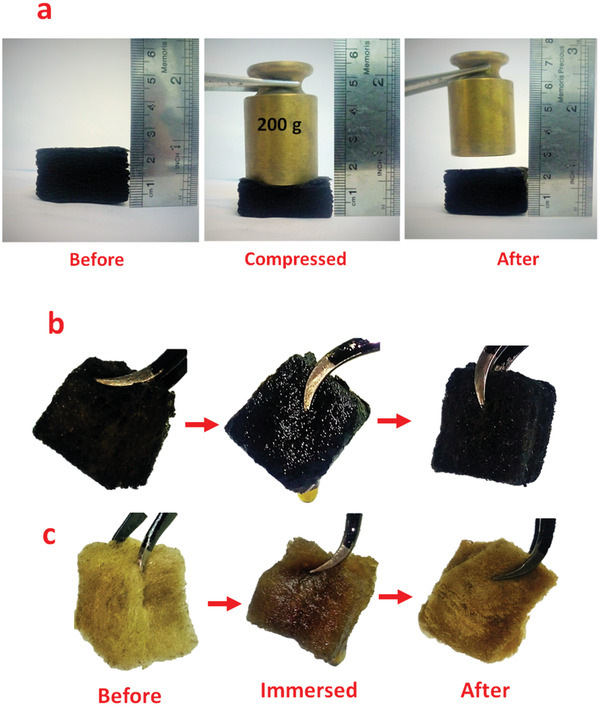
a) Mechanical strength test images of RGO/CR‐RW before and after loading a counterweight; b,c) appearance changes of modified and pristine RW after five squeezing cycles in engine oil.

To examine the stability of modified RW under multiple immersions and mechanical squeezing cycles, the mechanical properties of the modified RW were determined. **Figure** **5**a shows that the modified RW is compressed with 200 g weight (repeated 30 times). After removing the weight, the original shape of the RW was restored. Compressive strain efficiency (η′, %) is calculated by the following equation^[^ ^28^ ^]^

(1) $$\begin{array}{c} \eta^{\prime }=\frac{\left(H_{0}-H_{C}\right)}{H_{0}} \end{array}$$

where *H*_0_ represents the height of the original RW; *H*_C_ is the height of the compressed RW. The modified RW exhibited the compressive strain efficiency of 50% confirming that the modified RW is remarkably flexible. This is mainly due to the existence of CR adhesive materials on RW structure. Also, the composition of CR with RGO could further improve the mechanical properties of RW.

After several cycles of adsorption–desorption, the modified RW showed good recycling performance. Figure 5b,c shows that the modified RW has maintained its shape in high viscosity oil after five squeezing cycles, whereas the pristine RW shows severe mechanical damage by a simple squeezing due to its brittle nature. Ultimately, the shape of the modified RW returned to its normal size after the drying process. Therefore, RGO/CR‐RW is a suitable choice for multiple oil adsorption–squeezing cycles.

**Figure 6:**
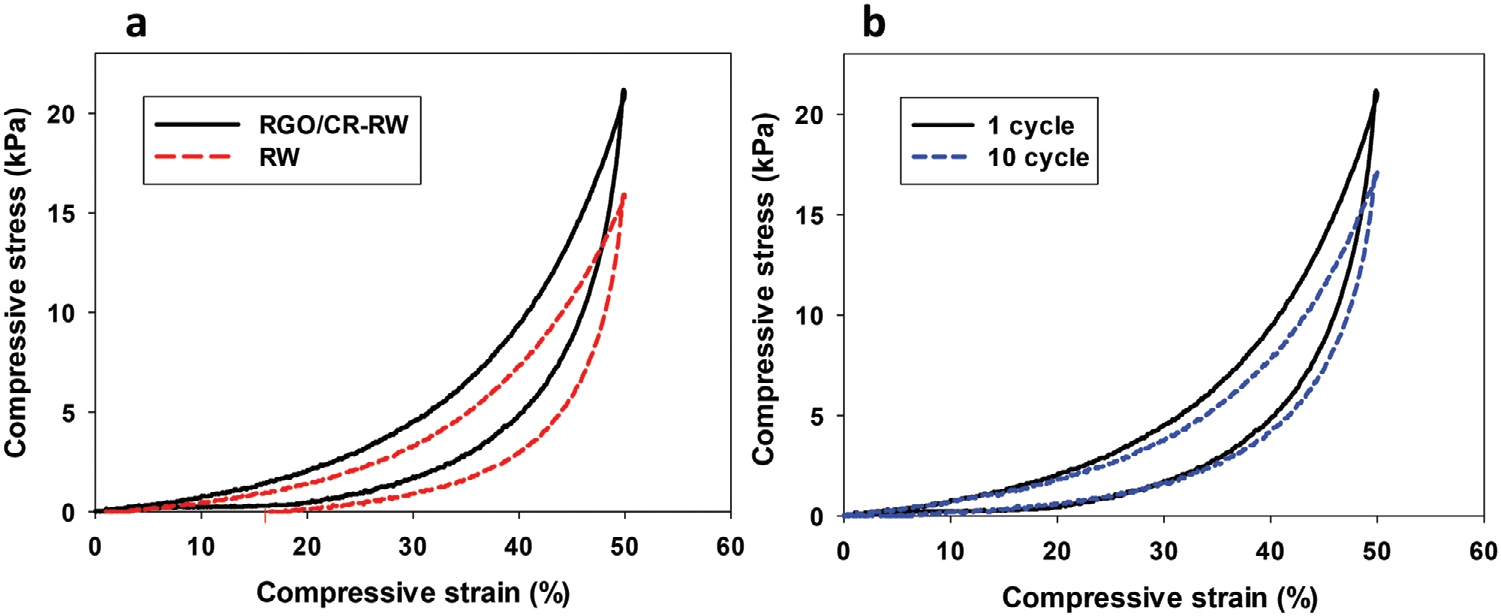
a) Cyclic compression curve of RW and RGO/CR‐RW at 50% strain. b) Cyclic compression curve of RGO/CR‐RW.

To evaluate the compressive durability and cyclic properties of the RW and RGO/CR‐RW, cyclic compression testing at 50% strain was conducted (**Figure** **6**). The modified RW completely restored to its original state after the external force was removed, indicating its great compressive durability and cyclic properties. However, pristine RW cannot be completely returned to its original height (Figure 6a). It shows about 70% recyclability. Figure 6b shows that the maximum compressive stress decreased from 21.4 to 17.3 kPa after ten cycles, indicating excellent mechanical robustness of the modified RW. Also, the unloading curve could return to the initial point after ten cycles, which suggested that the modified RW possessed outstanding elasticity. This performance makes the modified RW as a recyclable adsorbent for oil and organic solvents by manual squeezing.

### 2.5 Oil Adsorption and Oil–Water Separation

**Figure 7:**
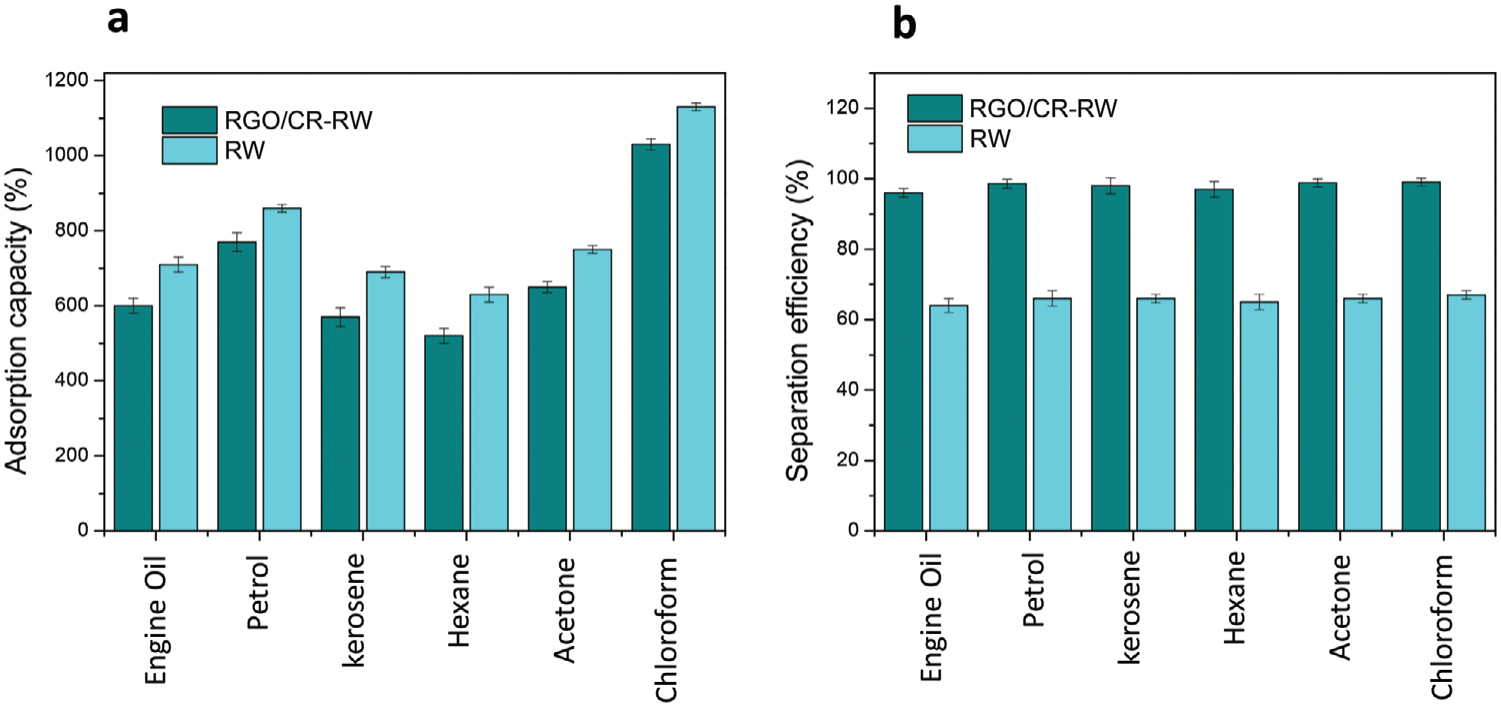
a) Adsorption capacity of RW and RGO/CR‐RW in several types of oils and organic solvents; b) separation efficiency of RW and RGO/CR‐RW in several types of oils and organic solvents.

The high porosity of RW makes it a promising adsorbent for a wide range of oils and organic solvents. **Figure** **7**a shows the adsorption capacity of the pristine and modified RW for various types of oils and organic solvents, including chloroform, acetone, hexane, petrol, kerosene, and engine oil. The adsorption capacity of modified RW was lower than that of pristine RW because the density of modified RW is much larger than the density of the pristine RW. According to **Table** **1**, the density and porosity of modified RW are 266.0 kg m^−3^ and 89.3%, respectively [calculated using Equation (3)]. Modification of RW significantly increased its weight. The higher density of the modified RW reduced its porosity. Chen et al.^[^ ^29^ ^]^ investigated the effect of melanin sponge density on its adsorption capacities. They also concluded that the adsorption capacity of modified melanin sponge was lower than that of the unmodified sponges due to higher density of modifier.

**Table 1:** Density and porosity of the RW and RGO/CR‐RW

Samples	ρ [kg m^−3^]	Φ [vol%]
RW	83.3	96.7
RGO/CR‐RW	266.0	89.3

Figure 7a also shows that the adsorption capacity of modified RW for organic solvents is higher than those of viscous oils such as engine oil. The modified RW exhibited the adsorption capacity ranges from 520 to 1030 wt%, depending on the density and viscosity of the oils and organic solvents. The adsorption capacities for liquids with lower density and higher viscosity were decreased. Low density liquids decrease the weight gain of the RW. Liquids with higher viscosity and surface tension resist their diffusion into porous sponges. These results are in agreement with previous reports.^[^ ^11^, ^30^ ^]^ It should be noted that the adsorption capacity of modified RW in the present work was much lower than those of several graphene‐based adsorbent materials such as graphene aerogels^[^ ^31^, ^32^ ^]^ and graphene/polyurethane sponges.^[^ ^14^, ^33^ ^]^ However, most of these adsorbent materials such as graphene aerogels for oil adsorption have high production cost and complex preparation processes. Low cost of the raw materials and easy fabrication of the modified RW will make it cost effective for scale‐up purposes. On the other hand, the modified RW used in this study exhibited greater performance when compared with some adsorbent materials reported in the literature such as PDMS sponges.^[^ ^30^, ^34^ ^]^ **Table** **2** lists the performance of the reported adsorbents compared to the modified RW in this work.

**Table 2:** Comparison of RGO/CR‐RW performance with reported adsorbent materials

Samples	Adsorption capacity [g g^−1^]	CA [°]	Cost	Ref.
PDMS‐graphene sponge	2.2–8.0	126.5	500 mg graphene: 100 USD 1 g PDMS: 23.7 USD 100 mL DMF: 20.5 USD https://www.sigmaaldrich.com Consumption rate: 50 mg graphene, 20 mL DMF, 40 g PDMS (specific surface area: 31 m^2^ g^−1^) Total: 962 USD ≈ 31.0 USD m^−2^ g^−1^	^[^ ^30^ ^]^
Graphene‐PU	29.0–33.0	152.0	500 mg graphene: 100 USD 250 g cellulose nanowhiskers: 37.6 USD 1 m^3^: 100 USD 1 EA PU (150 × 150 × 10 mm^3^): 84.7 USD https://www.sigmaaldrich.com Consumption rate: PU (40 × 40 × 20 mm^3^), 1 g graphene, 5 g cellulose nanowhiskers (surface area: 6.4 cm^3^) Total: 211.3 USD ≈ 33.0 USD cm^−3^	^[^ ^14^ ^]^
Graphene/MWCNT‐PDA aerogel	125.0–533.0	–	25 g graphite powder: 11.5 USD 1 L H_2_SO_4_: 73.7 USD 1 L H_2_O_2_: 6.4 USD 500 g NaNO_3_: 43.3 USD 25 g KMnO_4_: 18.10 USD 1 g MWCNT‐COOH: 127 USD 100 g PDA: 268 USD https://www.sigmaaldrich.com Consumption rate: 100 mg MWCNT‐COOH, 200 mg PDA, 50 mg GO (Surface area: 140 m^2^ g^−1^) Total: 13.23 USD ≈ 0.1 USD m^−2^ g^−1^	^[^ ^31^ ^]^
PDMS sponge	2.5–13.0	122.6 ± 1.7	25 g graphite powder: 11.5 USD 1 L H_2_SO_4_: 73.7 USD 1 L H_2_O_2_: 6.4 USD 500 g NaNO_3_: 43.3 USD 25 g KMnO_4_: 18.10 USD 500 g APTS: 126 USD 100 g DCC: 53.1 USD 100 g DMAP: 110 USD 1 g PDMS: 23.7 USD https://www.sigmaaldrich.com Consumption rate: 5 g PDMS, 0.2 g GO, 2.6 mL APTS, 4 g DCC, 0.3 g DMAP (surface area: 8 cm^3^) Total: 123 USD ≈ 15.4 USD cm^−3^	^[^ ^34^ ^]^
SiO_2_/PDMS‐RW	6.0–13.0	152.9	50 g SiO_2_ nanoparticles: 83.5 USD 100 mL PDMS‐OH: 68.6 USD 25 mL Tetraethoxysilane (TEOS): 28 USD 5 g Dibutyltin dilaurate (DBTDL): 15.2 USD https://www.sigmaaldrich.com 1 m^2^ RW: 1.8 USD https://www.alibaba.com Consumption rate: RW (4 × 4 × 2 cm^3^), 2 g PDMS‐OH, 0.4 g TEOS, 0.04 g DBTDL, 0.8 g SiO_2_ (Surface area: 64 cm^3^) Total: 50.7 USD ≈ 0.8 USD cm^−3^	^[^ ^13^ ^]^
RGO/CR‐RW	5.2–10.3	152.4 ± 0.2	25 g graphite powder: 11.5 USD 1 L H_2_SO_4_: 73.7 USD 1 L H_2_O_2_: 6.4 USD 500 g NaNO_3_: 43.3 USD 25 g KMnO_4_: 18.10 USD 250 g CR: 127 USD 250 mL toluene: 20.56 USD https://www.sigmaaldrich.com 1 m^2^ RW: 1.8 USD https://www.alibaba.com Consumption rate: RW (3 × 2 × 2 cm^3^), 2 mg GO, 300 µL CR, 40 mL toluene (Surface area: 42 cm^3^) Total: 3.2 USD ≈ 0.08 USD cm^−3^	Present work

The superhydrophobic and superoleophilic features of the modified RW are essential for selective oil adsorption in oil–water mixture. The oil–water separation efficiency (η, wt%) is calculated by the following equation^[^ ^28^ ^]^

(2) $$\begin{array}{c} \eta =\frac{m_{1}-m_{2}}{m_{3}} \end{array}$$

where *m*_1_ is the total weight of oil and water before separation; *m*_2_ is the weight of water after separation; and *m*_3_ is the weight of oil before separation. The oil–water separation efficiency was calculated to be above 95% for all oils and organic solvents with the ratio of oil to water 1:5 (Figure 7b). Separation efficiency for pristine RW was much lower than that of modified RW (an average 65% for oils and organic solvents). This indicates that the modified RW can effectively separate oil from water.

**Figure 8:**
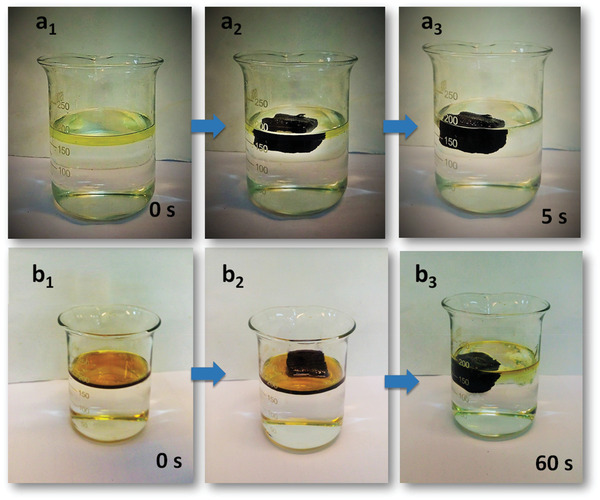
Adsorption process of a_1_–a_3_) petrol and b_1_–b_3_) engine oil floated on water with RGO/CR‐RW.

The adsorption rate of the RGO/CR‐RW composite is related to the solvents’ viscosity. It was really fast for low‐viscous oil and organic solvents. As shown in **Figure** **8**a_1_–a_3_, the petrol was immediately removed from the water surface by RGO/CR‐RW composite. The adsorption rate of RGO/CR‐RW composite in the same volume of high viscous engine oil was much lower and some excess oil remained on the water surface (Figure 8b_1_ **–**b_3_). The RGO/CR‐RW composite adsorbed large volume of viscous engine oil floated on the water surface within 60 s.

Video S2 (Supporting Information) demonstrates the separation of chloroform–water mixture by gravity. The chloroform was completely collected in an Erlenmeyer, while the water (dyed with methylene blue) was blocked in the balloon. No water could be seen in the Erlenmeyer. This indicates that the modified RW can separate the mixture of oil and water continuously.

### 2.6 Photothermal Properties

**Figure 9:**
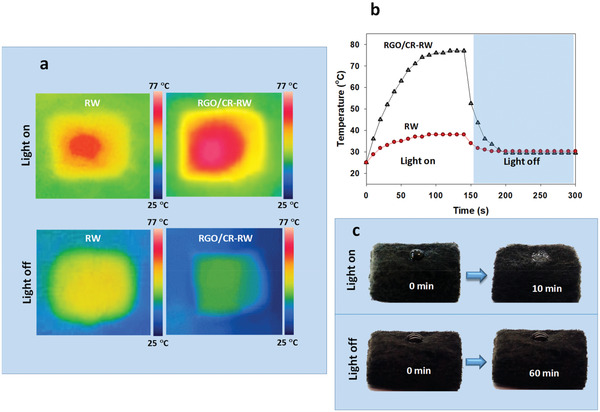
a) Thermal images of RW and RGO/CR‐RW under light on and off conditions; b) surface temperature changes of RW and RGO/CR‐RW with time under light on and off conditions; c) adsorption of a crude oil droplet on the surface of the modified RW under light on and off conditions (power density: 1.0 kW m^−2^ illumination).

Photothermal properties of RW and RGO/CR‐RW composite were compared to confirm the effectiveness of the modified RW in adsorption of highly viscous oil under natural sunlight irradiation. As the high‐viscosity oils are difficult to be adsorbed by the porous materials, increasing the temperature of the high‐viscosity oils can reduce their viscosity effectively.^[^ ^27^, ^35^ ^]^ With increasing the temperature, the viscosity of oil decreased and the oil adsorption rate increased.^[^ ^36^, ^37^ ^]^ Due to the outstanding thermal conductivity and effective photothermal properties corresponding to black RGO nanosheets,^[^ ^38^ ^]^ the modified RW can be heated up under illumination. The temperature changes of the pristine and modified RW were evaluated using a thermal infrared imager camera under illumination on (1.0 kW m^−2^) and off conditions (**Figure** **9**a). The surface temperature of modified RW increased rapidly under light illumination from 25 to 76 °C within 100 s. However, the temperature rise of pristine RW was lower than that of the modified RW. When light was off, the surface temperature of modified RW dropped faster than pristine ones which can be attributed to efficient heat dissipation (Figure 9b). The corresponding infrared thermal images of the pristine and modified RW under illumination showed that the modified RW is more effective in adsorption of highly viscous oil under natural sunlight. Figure 9c shows a highly viscous crude oil droplet on the surface of the modified RW. Under light illumination, the crude oil viscosity decreased and the modified RW completely adsorbed the crude oil after 10 min. The crude oil droplet remains almost unchanged without illumination for more than 60 min.

### 2.7 Recyclability

**Figure 10:**
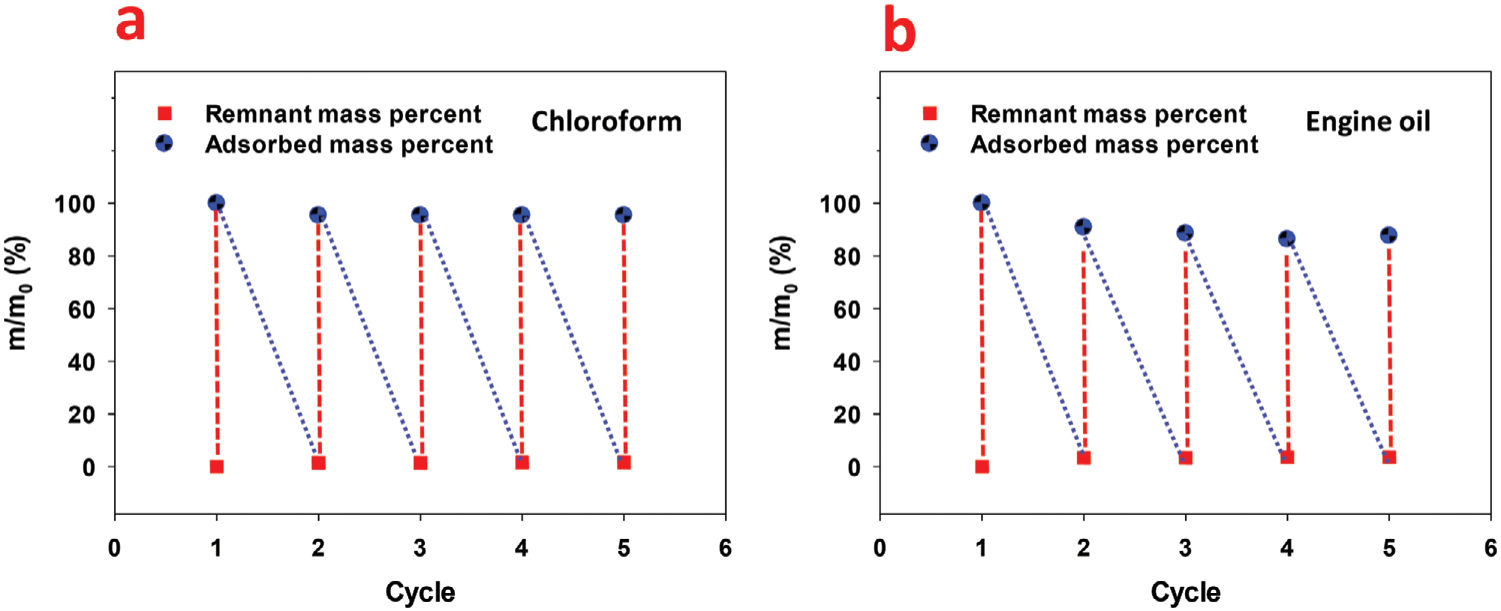
Recyclability of RGO/CR‐RW in a) chloroform and b) engine oil in five cycles.

The modified RW have a flexible structure, and thus squeezing is appropriate for its recycling. To investigate the recyclability of the RGO/CR‐RW composite, it was immersed in engine oil and chloroform and then squeezed to remove the adsorbed oil. This process was repeated several times. In order to remove residual oil, the RGO/CR‐RW composite was heated to decrease oil viscosity after squeezing. The chloroform was completely removed in each cycle. As **Figure** **10**a suggests, the adsorption capacity for chloroform did not change for five cycles and retained above 95% of the initial adsorption capacity. In the contrary, the adsorption capacity was declined in viscous engine oil after the first cycle because of the diffusion of oil within the RW pores (Figure 10b). The adsorption capacity of the RGO/CR‐RW composite in viscous engine oil up to the fifth cycle remained about 88% of the initial adsorption capacity. After five cycles of recycling, the adsorption capacity decreased. This may be attributed to the removal of some of RGO from the RW surface and slight damage of the surface structure in the repeated squeezing and drying process.

## 3 Conclusion

We have reported preparation of modified RW using RGO with excellent superhydrophobic property. Addition of CR into the porous structure of RW further enhanced the surface roughness and hydrophobicity. The RW without CR coating demonstrated severe mechanical damage, while the RW with CR coating exhibited excellent mechanical elasticity and durability. The modified RW exhibited the adsorption capacity from 520 to 1030 wt%, depending on the density and viscosity of the oils and organic solvents. Due to the higher density of modified RW, the adsorption capacity of modified RW is lower than that of unmodified one. We also showed that the adsorption rate with the high viscosity engine oil is lower than that of low viscosity oil. The modified RW efficiently separated oil/water mixtures with separation efficiency higher than 95%. Owning to easy modification procedure, low cost, high separation efficiency, excellent durability, and recyclability, the modified RW may be a promising candidate for removing oil spills on a large scale.

## 4 Experimental Section

### Materials

Rock wools were obtained from Pashme Sang Iran Co. (Mashhad, Iran). Polychloroprene adhesive was purchased from Shaya Chemi Ind. Co. Graphite powder (<0.1 mm, >95%) from Fluka; potassium permanganate (99%) from BDH; hydrogen peroxide (30%) from Fakhre Razi, sulfuric acid (95%), sodium nitrate (99%), hydrogen chloride (37%), barium chloride (99%), and silver nitrate (99.5%) from Merck; chloroform (98%), acetone (99%), toluene (95%), and hexane (97%) from Mojallali; engine oil from Behrang; petrol and kerosene from Persian Gulf Star Oil Co. were provided. All chemicals were used without further purification. Deionized (DI) water was used for preparation of solutions.

### Instruments

40 kHz ultrasonic cleaner YX‐2080 was used for dispersing graphite oxide. For drying the samples, the freeze dryer, model FD‐5010‐BT was used. The SEM images of the samples were recorded using Leo 1450VP. The FTIR spectra were obtained using Thermo Nicolet Avatar 370 FT‐IR Spectrometer scanning from 4000 to 500 cm^−1^. The XRD patterns were recorded using Bruker D8 Advanced diffractometer with Cu Kα radiation. The thermogravimetric analysis (TGA) was performed using BÄHR Thermal Analyzer model STA 503 to investigate the thermal stability of RW and RGO/CR‐RW sponges in the temperature range of 25–800 °C with a ramp rate of 10 °C min^−1^ under Ar atmosphere. The thermal images of the samples were obtained using a FLIR ONE thermal camera P/N 435‐0003‐01‐00 made. The WCA tests were measured using a commercial contact angle system of 5 V‐USB port power source equipped with a CCD camera and IrcA96 software for triplicate measurements. The volume of water droplet was 4 µL.

### Preparation of GO

GO was synthesized using modified Hummers’ method.^[^ ^39^ ^]^ Graphite powder (2 g) was gradually added to a beaker containing 70 mL concentrated H_2_SO_4_ in an ice bath. Then, 24 mmol NaNO_3_ was slowly poured while being stirred with a magnetic stirrer. Subsequently, 38 mmol potassium permanganate was gradually added into the mixture under stirring for 15 min with temperature control. The color of the mixture changed from brown to green due to the presence of oxidizing agent. After that, the ice bath was removed and the suspension stirred at room temperature for 48 h. Then, 100 mL DI water was added to the mixture and the suspension was further diluted by adding warm water (35 °C). To complete the oxidation process, 70 mL of H_2_O_2_ was added within 30 min. The product was centrifuged and washed several times with HCl (5 wt%) and then with DI water. To ensure the complete removal of SO_4_^2−^ and Cl^−^ ions, the effluent was tested using BaCl_2_ and AgNO_3_, respectively. The final product was freeze dried at −48 °C for 2 d.

### Preparation of Modified RW

In the first step, RW samples (3 × 2 × 2 cm^3^) were first cleaned with ethanol and deionized water and then dried. 300 µL of CR was dissolved in 40 mL toluene. A clean RW was placed in the solution for several minutes and then dried at room temperature. A layer of CR was adhered on the RW surface. In the second step, the RW sample was submerged in the GO dispersion (2 mg mL^−1^) for 1 h. Finally, the RW coated with GO sheets was reduced under vacuum at 200 °C for 2 h and RGO/CR‐RW was prepared. For comparative study, the original RW was submerged in the GO dispersion (2 mg mL^−1^) for 1 h followed by hydrothermal treatment under vacuum at 200 °C for 2 h and RGO/RW was prepared. The prepared RWs were dried at room temperature. Porosities of the RW samples were calculated using the following equation^[^ ^13^ ^]^

(3) $$\begin{array}{c} \phi =100\left(1-\frac{\rho_{a}}{\rho_{c}}\right) \end{array}$$

where ρ_a_ is the density of the sample and ρ_c_ is the density of granular quartz (2.5 g cm^−3^).

### Oil Adsorption Capacity and Recyclability

The adsorption capacity was determined by immersing RGO/CR‐RW into the 50 mL oils and organic solvents until full saturation. Then, it was weighed immediately after removing the surface oils or solvents. The adsorption capacity was calculated as follows^[^ ^13^ ^]^

(4) $$\begin{array}{c} Q_{m}\left(\text{g/g}\right)=\frac{\left(m_{1}-m_{0}\right)}{m_{0}} \end{array}$$

where *m*_0_ and *m*_1_ are the weights of the initial and saturated RW, respectively. The recyclability of the coated sample was tested by repeated adsorption–squeezing processes. The saturated sample was squeezed out by mechanical force and then heated to complete dryness. This adsorption–squeezing procedure was repeated five times. The weight of the sample before and after squeezing was recorded for each cycle.

## Conflict of Interest

The authors declare no conflict of interest.

## Supporting information

Supporting Information

Supplemental Video 1

Supplemental Video 2

## Data Availability

Research data are not shared.
