# Supplementary material for: A Mechanically Flexible Superhydrophobic Rock Wool Modified with Reduced Graphene Oxide‐Chloroperene Rubber for Oil‐Spill Clean‐Up
Source: Glob Chall. 2021 Sep 5;5(12):2100072. doi: 10.1002/gch2.202100072 (PMC8671620; doi:10.1002/gch2.202100072)
Supplement: Supplementary file 1 — Supporting Information [file GCH2-5-2100072-s001.pdf]

© 2021 Wiley-VCH GmbH

Global Challenges
Open Access

Supporting Information

for *Global Challenges,* DOI: 10.1002/gch2.202100072

A Mechanically Flexible Superhydrophobic Rock Wool Modified with Reduced Graphene Oxide-Chloroperene Rubber for Oil-Spill Clean-Up

*Maryam Davardoostmanesh and Hossein Ahmadzadeh**
